# Multiple Processes May Involve in the IgG4-RD Pathogenesis: An Integrative Study via Proteomic and Transcriptomic Analysis

**DOI:** 10.3389/fimmu.2020.01795

**Published:** 2020-08-20

**Authors:** Shaozhe Cai, Yu Chen, ShengYan Lin, Cong Ye, Fang Zheng, Lingli Dong

**Affiliations:** ^1^Department of Rheumatology and Immunology, Tongji Hospital, Tongji Medical College, Huazhong University of Science and Technology, Wuhan, China; ^2^Department of Immunology, School of Basic Medicine, Tongji Medical College, Huazhong University of Science and Technology, Wuhan, China; ^3^NHC Key Laboratory of Organ Transplantation, Ministry of Education, Chinese Academy of Medical Sciences, Wuhan, China

**Keywords:** IgG4-related disease (IgG4-RD), proteomic analysis, WGCNA (Weighted Gene Co-expression Network Analyses), enrichment analysis, IgG4-RD pathogenesis

## Abstract

Immunoglobulin G4-related disease (IgG4-RD) is a newly defined disease entity, while the exact pathogenesis is still not clear. Identifying the characters of IgG4-RD in proteomic and transcriptomic aspects will be critical to investigate the potential pathogenic mechanisms of IgG4-RD. We performed proteomic analysis realized with iTRAQ technique for serum samples from eight treatment-naive IgG4-RD patients and eight healthy volunteers, and tissue samples from two IgG4-RD patients and two non-IgG4-RD patients. Transcriptomic data (GSE40568 and GSE66465) was obtained from the GEO Dataset for validation. The weighted correlation network analysis (WGCNA) was applied to detect the gene modules correlated with IgG4-RD. KEGG pathway analysis was used to investigate pathways enriched in IgG4-RD samples. As a result, a total of 980 differentially expressed proteins (DEPs) in tissue and 94 DEPs in serum were identified between IgG4-RD and control groups. Three hundred fifty-four and two hundred forty-seven genes that most correlated with IgG4-RD were detected by WGCNA analysis in tissue and PBMC, respectively. We also found that DEPs in IgG4-RD samples were enriched in several immune-related activities including bacterial/viral infections and platelet activation as well as some immune related signaling pathways. In conclusion, we identified multiple processes/factors and several signaling pathways that may involve in the IgG4-RD pathogenesis, and found out some potential therapeutic targets for IgG4-RD.

## Introduction

Immunoglobulin G4-related disease (IgG4-RD) is a newly defined immune-mediated disease with common clinical, serological, and pathological features (1). Common features of IgG4-RD include serum IgG4 level elevation, multiple organ involvements, dense infiltration of IgG4+ plasma cells, and significant tissue infiltrates (2). This disease affects men more often than women and age at diagnosis ranges from 50 to 70 years (3). Although most patients do respond to steroids well, the relapse rate can be nearly 50% (4).

IgG4-RD is also characterized by alterations in acquired immune system, in which aberrant expansion of plasmablasts, CD4+ cytotoxic T cells, and follicular T helper cells have been observed (5–7). In addition, several inflammatory factors such as TGF-β, IL-4, and IL-10 have also been identified to play a role in the pathogenesis of IgG4-RD (8). The increase of these cytokines promotes eosinophilia in serum or certain tissues, high levels of IgG4-producing plasma cells, elevated production of IgE, and fibrosis, with inflammatory cell infiltrates ultimately causing organ damage (9). Furthermore, several autoantibodies, including anti-carbonic anhydrase II and anti-lactoferrin, are often present in patients with IgG4-RD, especially those with IgG4-related autoimmune pancreatitis (AIP) (10). At present, however, the exact pathogenic mechanism remains unclear, which is an important issue in IgG4-RD studies.

Due to the lack of ideal animal models, and limited sample origin, high throughput, and bioinformatics techniques may help understand the underlying pathogenesis of IgG4-RD more deeply. Transcriptome-wide profiling, as a downstream level of genome-scale mapping, can reveal a systemic dynamics of molecular interaction (11). Recently, studies have utilized transcript profiling in labial salivary glands (LSGs) to distinguish molecular features between IgG4-RD and Sjögren's syndrome (SS), a disease with common phenotypic elements (12, 13). Among other findings, active involvement of Th2- (IL-4, IL-5, and IL-21), T follicular helper cell (Tfh)—(BCL-6 and CXCR5) and Treg-related transcripts (IL-10, FOXP3, CCL18, and TGF-β1) in patients with IgG4-RD were observed. These data showed how elevated levels of such cytokines and chemokines can induce IgG4 plasma cell infiltration, high IgG4 levels in the periphery, and impact tissue fibrosis in the LSG of IgG4-RD patients (13). Further, researchers using high-throughput RNA sequencing technology revealed the molecular differences and effects from prednisone treatment among IgG4-related disease with salivary gland lesions (RD-SG), without SG lesions (RD-nonSG), and IgG4-related retroperitoneal fibrosis (RF) (14). However, the molecular mechanisms and appropriate therapeutic strategies underlying the pathogenesis of IgG4-RD are still unclear.

Proteins are effectors of biological function, and exert critical important roles in the pathogenesis of diseases. Investigation into proteins is crucial for the development of methods to realize early disease diagnosis, prognosis assessment and to monitor the disease development (15). Proteomics involves the applications of technologies for the identification and quantification of overall proteins present content of a cell, tissue or an organism. However, the proteomic study in the field of IgG4-RD is still blank. Thus, we detected both the serologic and tissue proteasome of IgG4-RD patients, and sought for the potential pathogenic information underlying the changes of expression level of proteins in IgG4-RD. Besides analysis at protein level, we also applied Weighted correlation network analysis (WGCNA) methods, a powerful analysis tool that can be utilized for constructing a weighted correlation network and finding modules comprised of highly correlated genes (16), to analyze two public datasets at transcriptomic levels in involved tissue and PBMC, respectively, of IgG4-RD patients.

In conclusion, based on proteomic and transcriptomic analyses, we have not only identified several differently expressed proteins in serum and tissue samples from IgG4-RD patients compared with healthy people, but also illustrated some features of immuno-inflammatory reactions in IgG4-RD, which also helped provide information of its potential therapeutic targets. These results may provide clues to the elucidation of the pathogenesis of, and the development of therapeutic agents for IgG4-RD.

## Materials and Methods

### Proteomic Analysis

#### Patients and Treatment

We studied eight diagnostic serum samples from eight treatment-naive IgG4-RD patients (25–70 years old) at Department of Rheumatology and Immunology, Wuhan Tongji Hospital (Table 1). The diagnosis of IgG4-RD has been made according to diagnostic criteria for IgG4-related disease (IgG4-RD) (17). Meanwhile, eight serum samples from healthy controls (HC) were collected and stored at −80°C for further analysis. Tissue samples of submandibular glands were obtained from two IgG4-RD patients and two non-IgG4-RD patients (adjacent normal edge of the surgical specimens), and stored in liquid nitrogen. All patients gave informed consent to the use of data records for research and to additional laboratory analysis on serum and tissue samples.

**Table 1:** Basic clinical information of IgG4-RD patients in this study.

**ID**	**Age**	**Involved organ**	**Sample type**
1	40–50	Retroperitoneum, pancreas, biliary tract, submandibular glands	Serum, tissue
2	40–50	Submandibular glands, retroperitoneum	Serum
3	50–60	Pancreas, biliary tract, submandibular glands, lymph nodes	Serum
4	50–60	Pancreas, biliary tract, submandibular glands	Serum, tissue
5	70–80	Pancreas, submandibular glands, salivary glands, lymph nodes	Serum
6	40–50	Pancreas, biliary tracts, lymph nodes	Serum
7	20–30	Endocranium, lymph nodes	Serum
8	50–60	Submandibular glands, lymph nodes	Serum

#### Sample Preparation

In this study, the Isobaric tags for relative and absolute quantitation (iTRAQ) technology was applied to investigate the proteasome of serum and tissue samples. First, the ProteoMiner Protein Enrichment Kit (Bio-rad laboratories, Hercules, CA, USA) was applied to deplete the high abundance proteins. Then, the protein solution (100 ug) with 8 M urea was diluted 4 times with 100 mM TEAB buffer. Trypsin Gold (Promega, Madison, WI, USA) was used to digest the proteins with the ratio of protein: trypsin = 40: 1 at 37°C overnight. After trypsin digestion, peptides were desalted with Strata X C (Phenomenex), and vacuum-dried according to the manufacturer's protocol. Peptide labeling was performed by iTRAQ Reagent. Peptide Fractionation were realized with a HPLC Pump system (Shimadzu LC-20AB) coupled with a high pH RP column. Supernatants of fractions were loaded on UHPLC system (Thermo Scientific™ UltiMate™ 3000) equipped with a trap and an analytical column, and peptides separated from nanoHPLC were subjected into the tandem mass spectrometry QEXACTIVE HF X (Thermo Fisher Scientific, San Jose, CA) for data-dependent acquisition (DDA) detection by nano-electrospray ionization. The parameters for MS analysis are listed as following: electrospray voltage: 2.0 kV; precursor scan range: 350–1,500 m/z at a resolution of 60,000 in Orbitrap; MS/MS fragment scan range: >100 m/z at a resolution of 15,000 in HCD mode; normalized collision energy setting: 30%; dynamic Exclusion time: 30 s; automatic gain control (AGC) for full MS target and MS2 target: 3e6 and 1e5, respectively; the number of MS/MS scans following one MS scan: 20 most abundant precursor ions above a threshold ion count of 10,000.

#### Protein Identification and Quantification

Protein identification and quantification were realized by software IQuant (18). The propensity score matchings (PSMs) were pre-filtered with false discovery rate (FDR) ≤ 1% to assess the confidence of peptides. Then, the identified peptide sequences were assembled into proteins. After protein inference, the protein will be estimated with FDR ≤ 0.01.

### Transcriptomic Analysis

#### Microarray Data Collection

We downloaded DNA microarray dataset GSE40568 and GSE66465 from Gene Expression Omnibus (GEO). Specifically, LSG samples in GSE40568 were obtained from Japanese patients with IgG4-RD (*n* = 5) as well as from Japanese patients with SS (*n* = 5) and HCs (*n* = 3) who had been followed up at the University of Tsukuba Hospital (Ibaraki, Japan), Tokyo Women's Medical University Hospital (Tokyo, Japan), and Kyushu University Hospital (Fukuoka, Japan) (13). PBMC samples from peripheral blood mononuclear cell (PBMC) of IgG4-RD were obtained from patients with IgG4-RD before (*n* = 2) and after steroid (*n* = 2) therapy who registered in the research project of the Research Program for Intractable Disease of the Ministry of Health, Labor, and Welfare (MHLW) of Japan and HCs (*n* = 4) (19).

#### WGCNA Analysis

The coefficient of variation (CV) of each gene were calculated after expression matrix were imported and normalized. Genes with CV >5% were log2 transformed, and the corresponding expression data was applied as input for WGCNA analysis. Then weighted co-expression networks were constructed by employing blockwiseModules function in the WGCNA package (https://horvath.genetics.ucla.edu/html/CoexpressionNetwork/Rpackages/WGCNA/). In this study, we construct a scale-free network (*R*^2^ = 0.9) based on the criteria that soft-thresholding power β were set as 20 (IgG4-RD LSG samples in GSE40568 dataset, Figure S1) and 12 (IgG4-RD PBMC sample in GSE66465 dataset, Figure S2) correspondingly. Genes, that possess edges with adjacency value of >0.2 in the module most correlated to IgG4-RD, were extracted for enrichment analysis.

#### Statistical Analysis

Log2 transformed data were used to calculate the difference of proteins between IgG4-RD and HC samples with “*t*-test” function in R package (version 3.5.1). Expression matrix from GEO datasets were extracted and normalized by using R package “GEOquery”. WGCNA analysis was realized with R package “WGCNA” (version 1.68) (16). KEGG/GO analyses and network construction were realized and visualized with Cytoscape (version 3.4.0) and ClueGO plugin or R package “clusterprofiler” (version 3.12.0) (20, 21). Information of targeted drugs of hub proteins were obtaind via Therapeutic Target Database (22). Without specific indication in the manuscript, all DEPs or genes in gene-module were input to make enrichment analyses.

## Results

### Identification of Differentially Expressed Proteins With Proteomic Data

**Figure 1:**
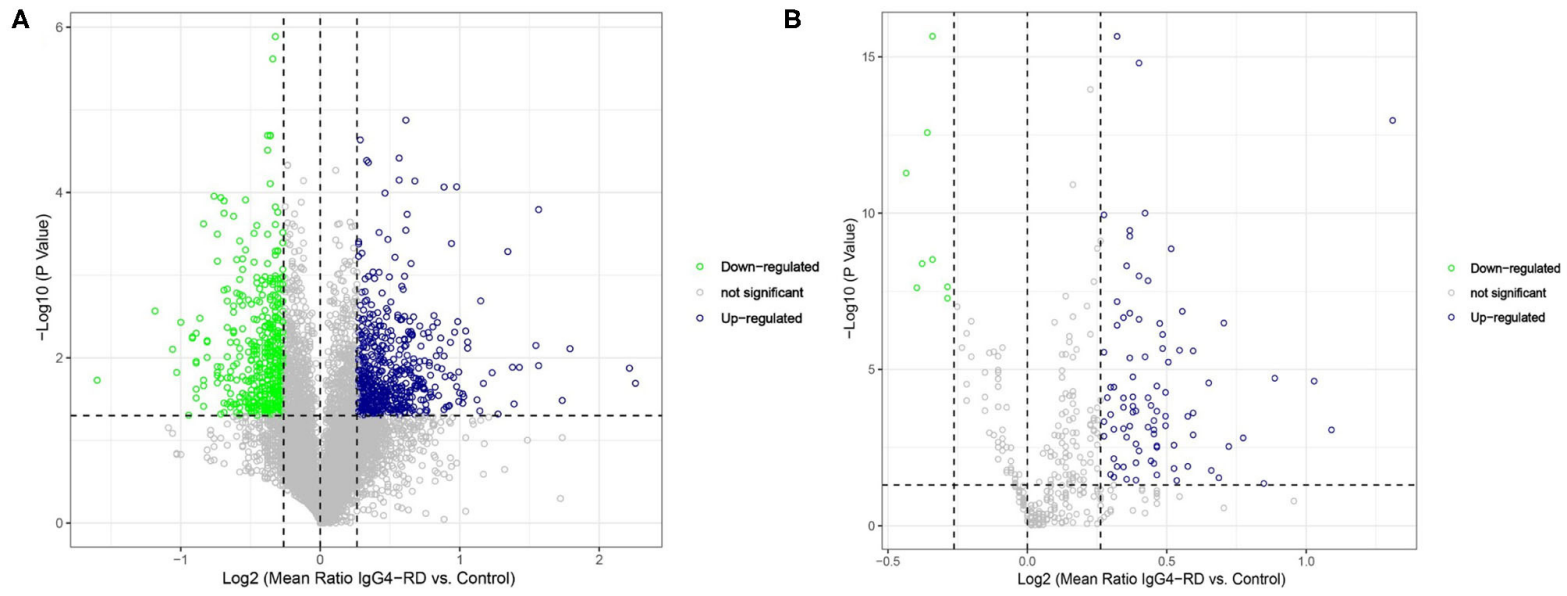
Volcano plot of differentially expressed proteins between IgG4-RD patients and control. **(A)** 980 (542 up-regulated, 438 down-regulated) differentially expressed proteins were identified in tissue between IgG4-RD and control samples. **(B)** 94 (86 up-regulated, 8 down-regulated) proteins in serum were identified as differentially expressed between IgG4-RD and control samples. Proteins with mean ratio >1.2 and *p* < 0.05 were regarded as differentially expressed.

A total of 980 (542 up-regulated and 438 down-regulated) differentially expressed proteins (DEPs) in tissue (Figure 1A, Table S1), while 94 (86 up-regulated, 8 down-regulated) differentially expressed proteins in serum (Figure 1B, Table S2) were identified between IgG4-RD and control samples based on our criteria (mean ratio of IgG4-RD vs. Control ≥1.2, *p* < 0.05). Among them, we found there were 12 DEPs (IGHG4, ITA2B, URP2, HV118, APOC2, GP1BA, CAP1, TBB1, APOE, DSC2, TSP1, and SODE) overlapped in the comparisons of tissue and serum and all those DEPs upregulated in IgG4-RD patients, suggesting their importance to IgG4-RD.

**Figure 2:**
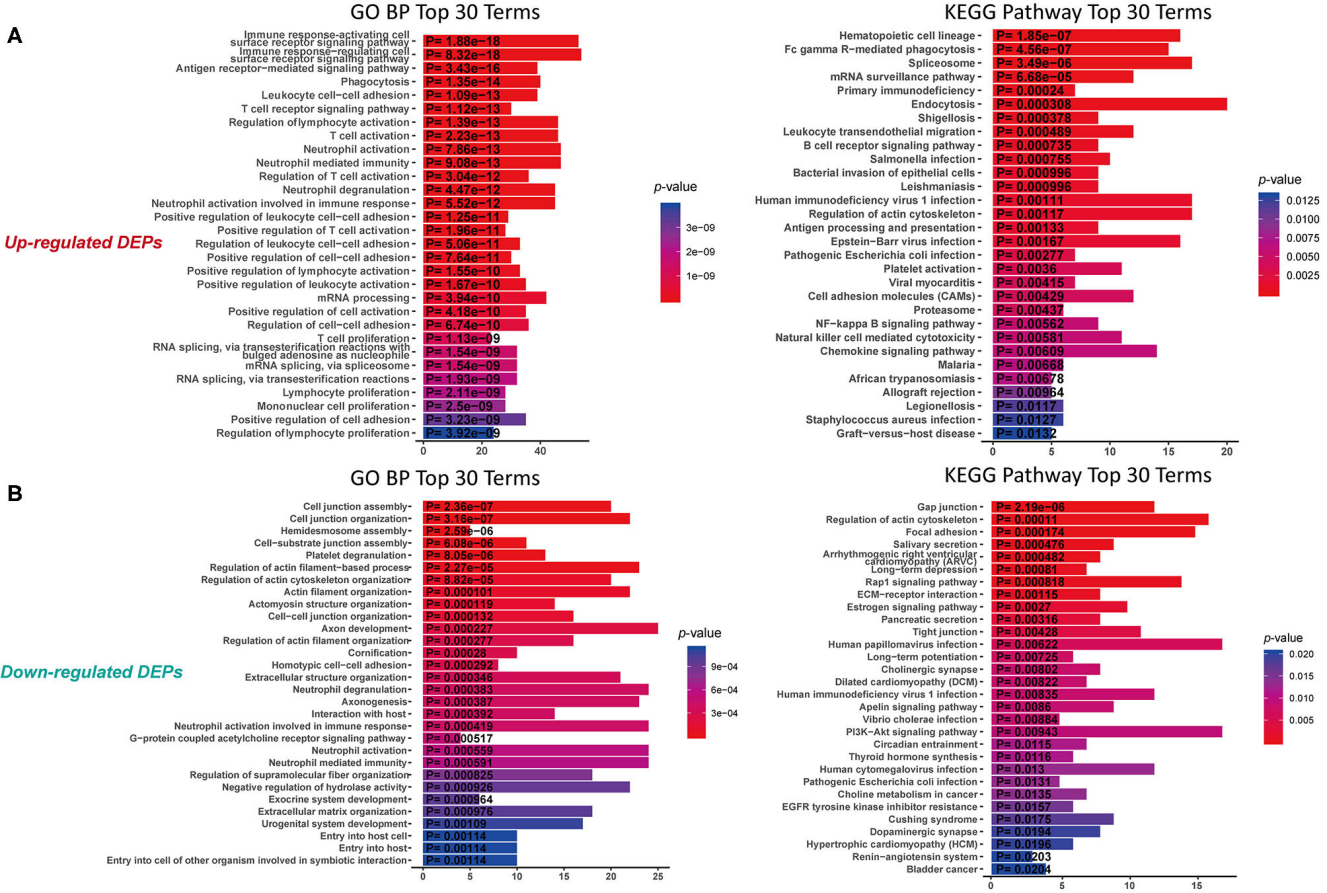
Functional enrichment of DEPs in tissue with proteomic data. **(A)** KEGG analysis and GO Biological process (GO BP) enrichment of up-regulated DEPs in tissue. **(B)** KEGG analysis and GO enrichment of down-regulated DEPs in tissue. X-axis: the number of DEPs of the proteomic data involved in the corresponding enriched terms.

**Figure 3:**
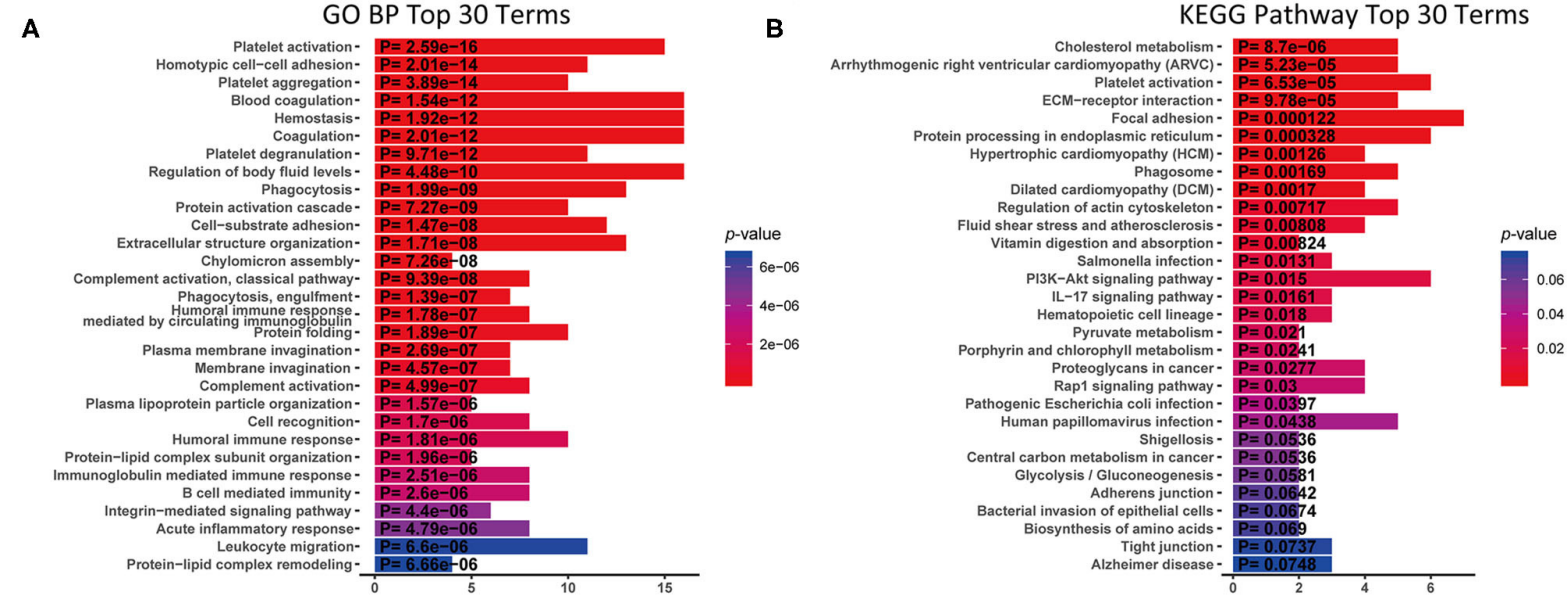
KEGG analysis and GO Biological process (GO BP) enrichment of up-regulated DEPs in serum with proteomic data. X-axis: the number of DEPs of the proteomic data involved in the corresponding enriched terms. **(A)** GO BP enrichment analysis. **(B)** KEGG pathway analysis.

To understand the function of these DEPs which may involve in IgG4-RD, functional enrichment analysis of these DEPs identified in tissue/serum between IgG4-RD and control samples were carried out. Results indicated that most tissue upregulated DEPs were involved in terms including immune related cells activation (e.g., immune response-activating cell surface receptor signaling pathway) and cell adhesion (e.g., leukocyte cell-cell adhesion), and infection related processes such as human immunodeficiency virus 1 infection, Epstein-Barr virus infection, and Salmonella infection etc. (Figure 2A). However, down-regulated DEPs in tissue were mainly involved in processed related to cell junction (e.g., cell junction assembly) (Figure 2B). Given the number of downregulated DEPs in serum is small, we focus on upregulated DEPs for further analysis and terms such as protein activation cascade, platelet activation, and extracellular structure organization were outstood in both GO biological process (BP) and KEGG pathway (Figure 3).

### Weighted Gene Co-expression Network Analysis of Transcriptomic Data

**Figure 4:**
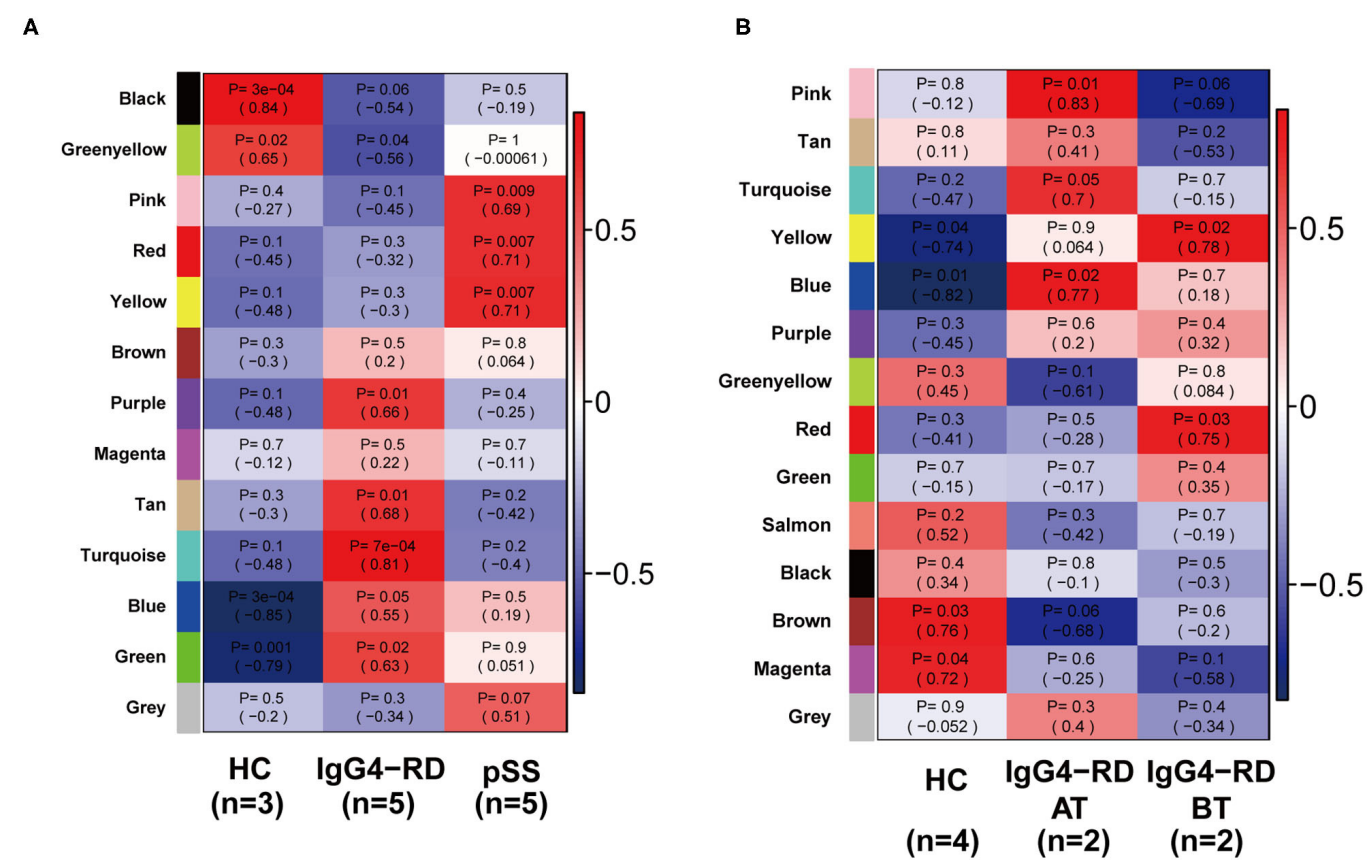
Heatmap of module-trait relationships revealed by WGCNA analysis. **(A)** 13 modules were detected by WGCNA in all samples from GSE40568. Among all these 13 modules, module “turquoise” showed strongest correlation with IgG4-RD phenotype. **(B)** 14 modules were detected by WGCNA in all samples from GSE66465, and module “yellow” showed strongest correlation with IgG4-RD (before treatment) phenotype.

A total of 4,364 genes with coefficient of variation (CV) >5% as input for the construction of WGCNA using GSE40568 dataset from LSC samples. Combined with the topological overlap matrix with the hierarchical average linkage clustering method, we detected the 13 modules in IgG4-RD (*n* = 5), pSS (*n* = 5), and HC samples (*n* = 3) (Figure 4A). Among them, module “turquoise” with 934 genes showed strongest correlation with IgG4-RD phenotype (correlation = 0.81, *p* = 7e-5) (Figure 4A, Figures S3A,B). Then, a total of 354 genes with edge-adjacency-value more than 0.2 in module “turquoise” were exported for further functional enrichment analysis (Tables S3, S4).

Based on GSE66456 dataset from PBMC samples, we identified 2,306 genes with coefficient of variation (CV) >5% as input for WGCNA. In this study, 14 modules were detected by WGCNA in IgG4-RD before treatment (IgG4-RD_BT, *n* = 2), IgG4-RD after treatment (IgG4-RD_AT, *n* = 2) and HC samples (HC, *n* = 4) based on the criteria referred in method section (Figure 4B). Among them, module “yellow” with 360 genes showed strongest correlation with IgG4-RD_BT phenotype (Figure 2B, Figures S4A,B). Further, 247 genes with edge-adjacency-value more than 0.2 in module “yellow” were exported for functional enrichment (Tables S5, S6). In general, we discovered three major biological processes and several signaling pathways that may involve in IgG4-RD.

### Antibody Mediated Autoimmune Responses Are the Character of IgG4-RD

Antigen-receptor mediated signaling pathway was significantly enriched in IgG4-RD tissues. Besides T cell-related signaling pathways, using proteomic data by KEGG analysis, we found several KEGG pathways related to B cell-related immune processes, such as “Fc gamma R-mediated phagocytosis,” “B cell receptor signaling pathway,” “Antigen processing and presentation,” and “Leukocyte transendothelial migration” were significantly enriched in IgG4-RD tissue (Figure 2A). Meanwhile, we also found term like “Fc gamma R-mediated phagocytosis” was enriched in genes from “turquoise” module in WGCNA analysis of the transcriptomic data of IgG4-RD LSG (Table S7). In addition, KEGG term “Systemic lupus erythematosus” was enriched in transcriptomic data of IgG4-RD derived PBMC (*p* < 0.0001, Table S8). These results indicated autoimmune characters of IgG4-RD, and also revealed an important role of antibodies in the autoimmune responses of IgG4-RD (23).

### Potential Infection and Infection-Related Responses May Be the Trigger in IgG4-RD

Infectious agents are the main origin of pathogen associated molecular patterns (PAMPs), and can mediate the release of danger associated molecular patterns (DAMPs). Both PAMPs and DAMPs are ligands of pattern recognition receptors (PRRs), which can be found in many cellular components involved in immune reactions. Activation of PRRs can modulate the functional states of immune-related cells, which further influence the process of immune responses. Based on our proteomic data, many infection related terms such as “Human immunodeficiency virus 1 infection,” “Epstein-Barr virus infection” and “Bacterial invasion of epithelial cells” were enriched in IgG4-RD tissue (Figure 2A, Table 2). Meanwhile, we also found genes from module “turquoise” in transcriptomic data also showed correlation with infection terms such as “Human papillomavirus infection,” “Epithelial cell signaling in Helicobacter pylori infection,” “Bacterial invasion of epithelial cells,” etc (Table 3, Table S7). Most of these terms showed relationship with bacterial infection, which may echo the fact that most involved organs in IgG4-RD are exocrine organs, like pancreas, and submandibular glands (24). Thus, we may infer that infectious agents and infection related processes may play important roles in the development and progress of IgG4-RD.

**Table 2:** KEGG terms related to infectious process enriched in IgG4-RD tissue proteomic data.

**Pathway ID**	**KEGG term**	**Associated proteins**	***P*-value**
KEGG:05143	African trypanosomiasis	FAS, GNAQ, IL18, LAMA4, PLCB1, PLCB2, PRKCB, VCAM1	0.0010
KEGG:05146	Amoebiasis	CASP3, GNA11, GNAQ, LAMA4, LAMB3, PLCB1, PLCB2, PRKACB, PRKCB, RAB5C, SERPINB9, TLR2	0.0159
KEGG:05100	Bacterial invasion of epithelial cells	ARPC1B, ARPC2, ARPC3, ARPC5, CDH1, DNM3, ELMO1, ELMO3, RHOG, SEPT1, WAS	0.0050
KEGG:05142	Chagas disease American trypanosomiasis	C1QB, CD3E, CD3G, FAS, GNA11, GNAO1, GNAQ, PLCB1, PLCB2, PPP2CB, PPP2R1B, TLR2	0.0331
KEGG:05120	Epithelial cell signaling in Helicobacter pylori infection	ADAM17, ATP6V0C, ATP6V1G2, CASP3, CSK, F11R, LYN, NOD1, PLCG2, TJP1	0.0079
KEGG:05170	Human immunodeficiency virus 1 infection	AP1G2, AP1S3, APOBEC3C, APOBEC3F, ATM, CASP3, CD3E, CD3G, CD4, CFL1, CFL2, FAS, GNA11, GNAO1, GNAQ, GNG2, GNG7, HLA-E, LIMK1, MAP2K1, PLCG2, PRKCB, RAC2, RPS6KB2, SAMHD1, TLR2, TRADD, TRIM5	0.0001
KEGG:05134	Legionellosis	CASP3, CR1, HSF1, HSPA6, IL18, PYCARD, RAB1A, TLR2	0.0177
KEGG:05140	Leishmaniasis	CR1, CYBB, HLA-DQA2, HLA-DQB1, HLA-DRA, ITGA4, PRKCB, PTPN6, TLR2	0.0447
KEGG:05144	Malaria	CR1, IL18, ITGAL, SDC2, THBS1, TLR2, VCAM1	0.0282
KEGG:05130	Pathogenic *Escherichia coli* infection	ARPC1B, ARPC2, ARPC3, ARPC5, CDH1, EZR, KRT18, TUBA4A, TUBAL3, TUBB1, TUBB4B, WAS	0.0001
KEGG:05132	Salmonella infection	ARPC1B, ARPC2, ARPC3, ARPC5, IL18, KLC3, KLC4, PFN1, PKN1, PYCARD, RHOG, TJP1, WAS	0.0020
KEGG:05131	Shigellosis	ARPC1B, ARPC2, ARPC3, ARPC5, ELMO1, ELMO3, NOD1, PFN1, RHOG, UBE2D2, WAS	0.0018
KEGG:05110	Vibrio cholerae infection	ATP6V0C, ATP6V1G2, PLCG2, PRKACB, SLC12A2, TJP1, TJP2	0.0311
KEGG:05416	Viral myocarditis	CASP3, CXADR, DAG1, DMD, HLA-DQA2, HLA-DQB1, HLA-DRA, HLA-E, ITGAL, RAC2	0.0028

**Table 3:** KEGG terms related to infectious process enriched in “turquoise” module in IgG4-RD tissue transcriptomic data.

**Pathway ID**	**KEGG term**	**Associated proteins**	***P*-value**
KEGG:05165	Human papillomavirus infection	AKT3, COL1A1, COL1A2, COL6A1, COL6A3, EGFR, FN1, FZD7, ITGA9, JAG1, LAMA2, LAMA4, LAMC1, THBS2	0.0052
KEGG:05100	Bacterial invasion of epithelial cells	CAV1, CAV2, FN1, MET, WASF2	0.0112
KEGG:05146	Amoebiasis	COL1A1, COL1A2, COL3A1, FN1, GNAL, LAMA2, LAMA4, LAMC1	0.0004
KEGG:05144	Malaria	CD36, HGF, MET, SDC2, THBS2	0.0019

### Platelet Activation Were Observed in IgG4-RD Samples

**Figure 5:**
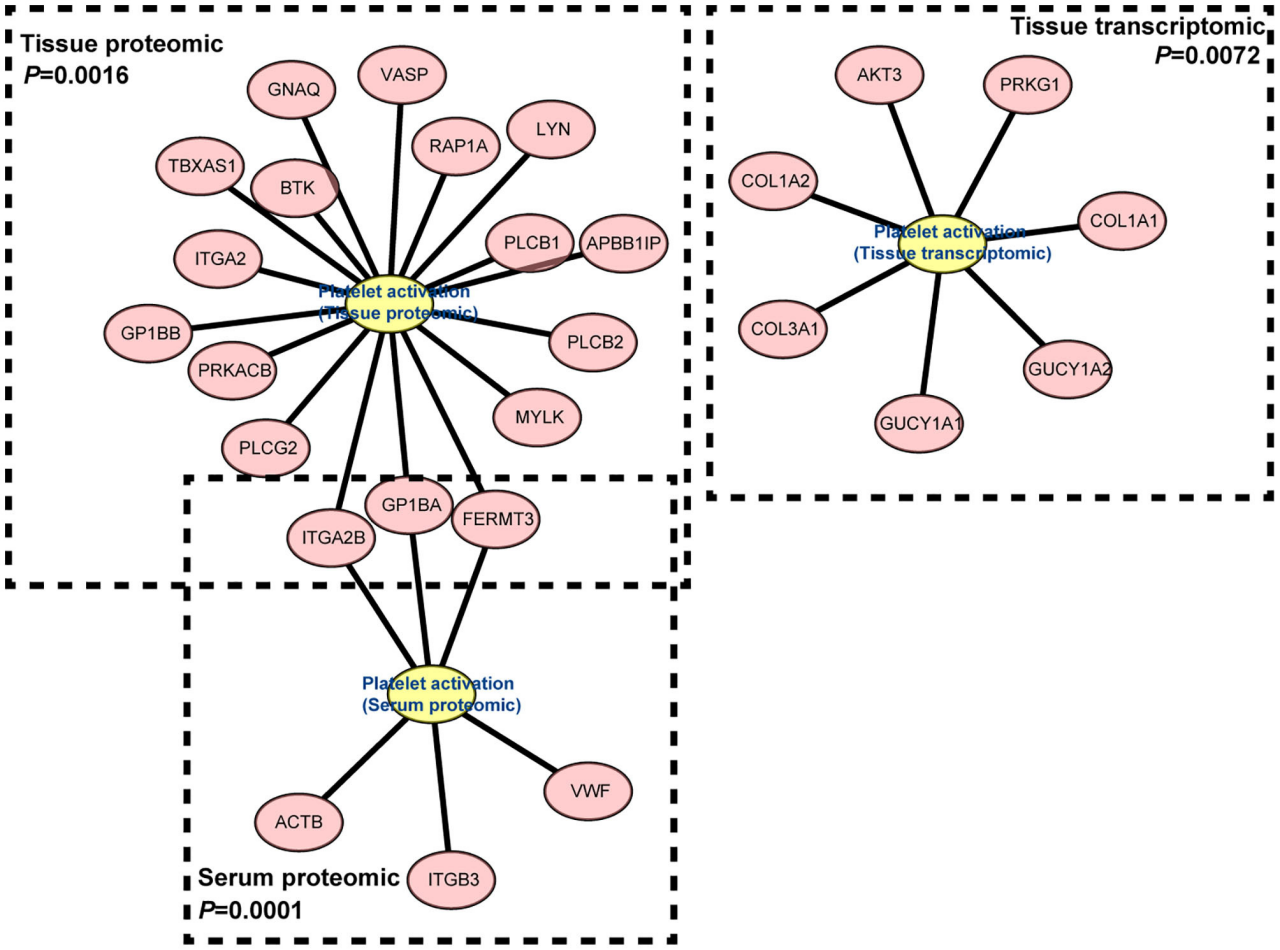
Gene network of “Platelet activation” term derived from “turquoise” module in IgG4-RD tissue transcriptomic dataset, proteomic data from tissue, and serum.

In proteomic data, up-regulated DEPs from both serum and tissue samples of IgG4-RD patients were enriched in “Platelet activation” pathway (Figures 2A, 3). Meanwhile, Enrichment of term “Platelet activation” was also observed in genes derived from “turquoise” module from tissue transcriptomic dataset (Figure 5). This result is consistent with the research on platelets that may serve as the immune components to possess modulate functions (25).

### Multiple Signaling Pathways May Participate the Pathogenesis of IgG4-RD

**Figure 6:**
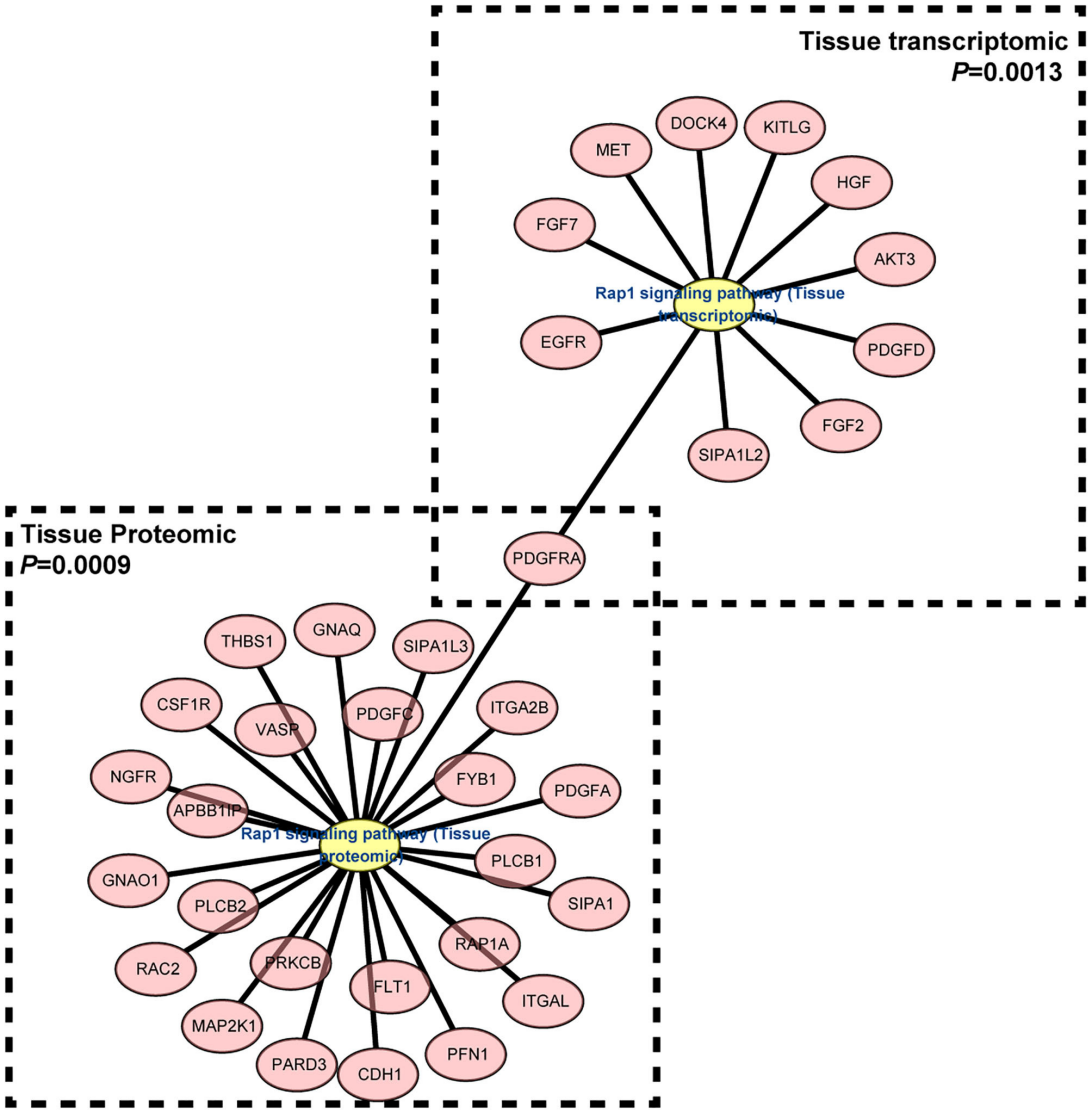
Gene network of “Rap1 signaling pathway” term enriched in both IgG4-RD tissue proteomic data and LSG transcriptomic dataset.

Understanding the significantly enriched signaling pathways in IgG4-RD may help to search the potential therapeutic targets. In proteomic data, “Rap1 signaling pathway” and “NF-κB signaling pathway” were enriched in IgG4-RD tissue (Figure 2B), while terms such as “MAPK signaling pathway,” “PI3K-Akt signaling pathway,” “TGF-β signaling pathway,” “Ras signaling pathway,” and “Rap1 signaling pathway” were enriched in tissue transcriptomic data (Table 4, Table S7). Term “TGF-β signaling pathway” was also enriched in transcriptomic data from IgG4-RD PBMC (Table S8). In this study, only “Rap1 signaling pathway” was observed enriched in both tissue proteomic data and IgG4-RD LSG samples in GSE40568 dataset (Figures 2B, 6). Activation of Rap1 signaling pathway can lead to the production of proinflammatory cytokines and modulate the expression level of MMPs, which are critical in the modulation of extracellular matrix, and influence the fibrogenic process (26). Beside terms illustrated above, there were also many other terms, like “Autophagy,” “Necroptosis,” etc., that were enriched in different datasets (Tables S7, S8).

**Table 4:** Signaling pathways and other potential pathogenic processes detected in IgG4-RD tissue transcriptomic data.

**Pathway ID**	**KEGG term**	**Associated proteins/genes**	***P*-value**	**Data origin**
KEGG:04350	TGF-beta signaling pathway	BMP6, LTBP1, SMAD1, THBS1	0.0409	PBMC transcriptomic
KEGG:04217	Necroptosis	HIST1H2AB, HIST1H2AE, HIST1H2AH, HIST1H2AI, HIST1H2AJ, HIST1H2AK, HIST1H2AL, HIST1H2AM, TNFAIP3	0.0009	PBMC transcriptomic
KEGG:05203	Viral carcinogenesis	HIST1H2BB, HIST1H2BC, HIST1H2BG, HIST1H2BH, HIST1H2BI, HIST1H2BJ, HIST1H2BM, HIST1H2BN, HIST1H2BO, HIST1H4D, HIST2H2BE, HIST2H4A	0.0001	PBMC transcriptomic
KEGG:05202	Transcriptional misregulation in cancer	CD14, HIST1H3A, HIST1H3B, HIST1H3F, HIST1H3H, HIST1H3J, MEIS1, SMAD1	0.0081	PBMC transcriptomic
KEGG:04064	NF-kappa B signaling pathway	ATM, BTK, LYN, PARP1, PLCG2, PRKCB, TNFRSF11A, TRADD, UBE2I, VCAM1, ZAP70	0.0471	Tissue proteomic
KEGG:04015	Rap1 signaling pathway	APBB1IP, CDH1, CSF1R, FLT1, FYB1, GNAO1, GNAQ, ITGA2B, ITGAL, MAP2K1, NGFR, PARD3, PDGFA, PDGFC, PDGFRA, PFN1, PLCB1, PLCB2, PRKCB, RAC2, RAP1A, SIPA1, SIPA1L3, THBS1, VASP	0.0009	Tissue proteomic
KEGG:04140	Autophagy	DAPK1, GABARAPL1, HMGB1, MAP2K1, MTMR14, PIK3R4, PPP2CB, PRKACB, RPS6KB2, RRAGA, RRAGC, STX17, ULK2, WIPI1	0.0371	Tissue proteomic
KEGG:04217	Necroptosis	CHMP1B, CHMP4C, CYBB, FAS, FTH1, FTL, H2AFX, H2AFY, H2AFY2, HIST2H2AB, HMGB1, PARP1, PYCARD, PYGM, TRADD, TYK2, ZBP1	0.0289	Tissue proteomic
KEGG:05219	Bladder cancer	CDH1, DAPK1, MAP2K1, RPS6KA5, THBS1, TYMP	0.0368	Tissue proteomic
KEGG:04010	MAPK signaling pathway	AKT3, EGFR, FGF2, FGF7, HGF, KITLG, MAP3K20, MET, NTRK2, PDGFD, PDGFRA, TGFBR2	0.0115	Tissue transcriptomic
KEGG:04015	Rap1 signaling pathway	AKT3, DOCK4, EGFR, FGF2, FGF7, HGF, KITLG, MET, PDGFD, PDGFRA, SIPA1L2	0.0013	Tissue transcriptomic
KEGG:04151	PI3K-Akt signaling pathway	AKT3, COL1A1, COL1A2, COL6A1, COL6A3, EGFR, FGF2, FGF7, FN1, GHR, GNG11, HGF, ITGA9, KITLG, LAMA2, LAMA4, LAMC1, MET, NTRK2, PDGFD, PDGFRA, THBS2	<0.0001	Tissue transcriptomic
KEGG:05226	Gastric cancer	AKT3, EGFR, FGF2, FGF7, FZD7, HGF, MET, TGFBR2	0.0059	Tissue transcriptomic
KEGG:05222	Small cell lung cancer	AKT3, FN1, LAMA2, LAMA4, LAMC1	0.0276	Tissue transcriptomic
KEGG:05215	Prostate cancer	AKT3, EGFR, PDGFD, PDGFRA, PLAT	0.0323	Tissue transcriptomic
KEGG:05205	Proteoglycans in cancer	AKT3, ANK2, CAV1, CAV2, COL21A1, DCN, EGFR, FGF2, FN1, FZD7, GPC3, HGF, LUM, MET, SDC2, TIMP3	<0.0001	Tissue transcriptomic
KEGG:05218	Melanoma	AKT3, EGFR, FGF2, FGF7, HGF, MET, PDGFD, PDGFRA	<0.0001	Tissue transcriptomic

### Potential Therapeutic Target Identified by Tissue DEPs Related Biological Processes

**Figure 7:**
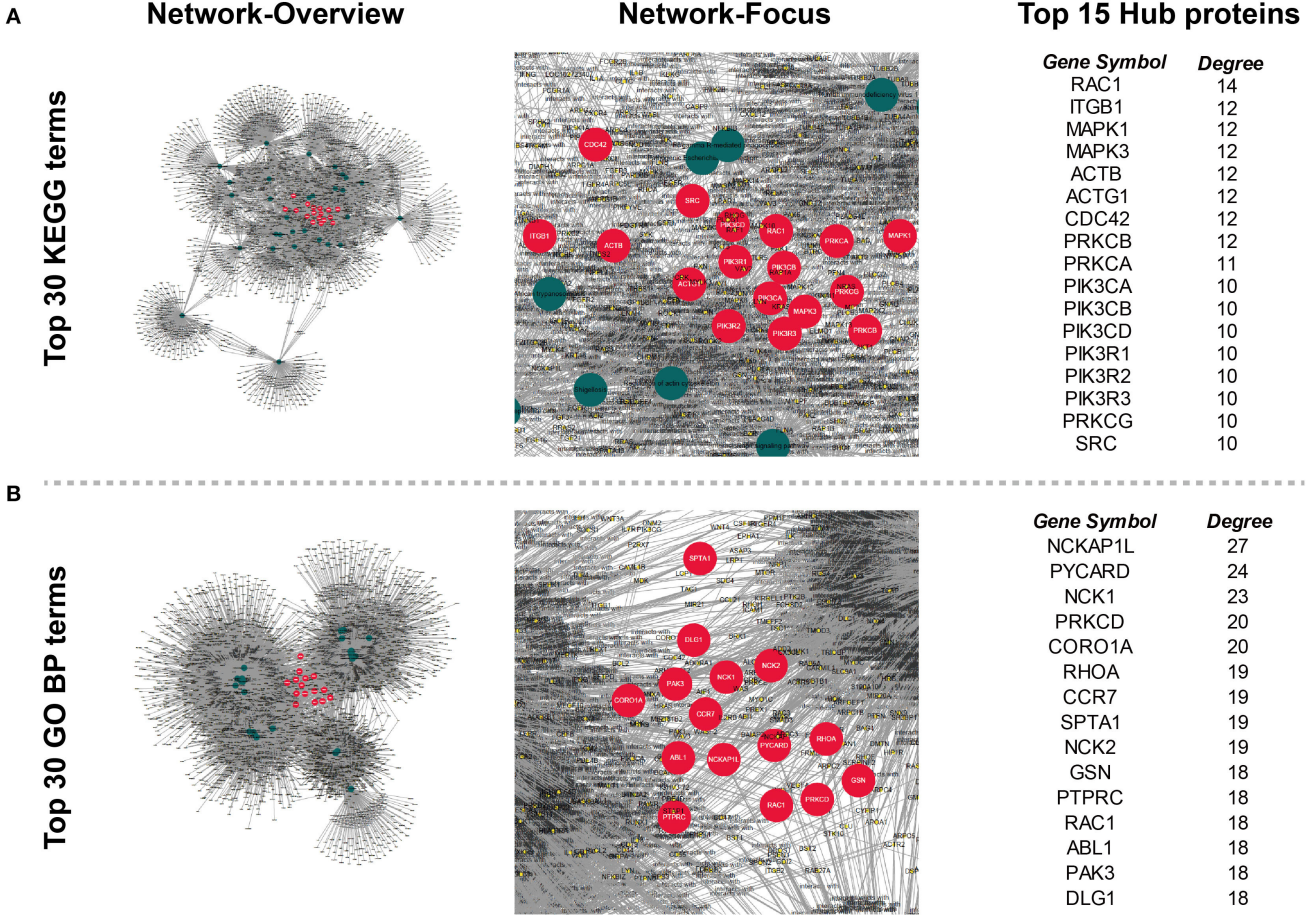
Networks constructed by all proteins involved in the top 30 **(A)** KEGG and **(B)** GO Biological process (GO BP) terms enriched from all tissue DEPs. Circles (nodes) in the network represents proteins or KEGG/GO terms. Degree (number of connection) of each node was calculated, and nodes (except that represent KEGG/GO terms) with top 15 degree were regarded as hub nodes, and their corresponding proteins were identified as hub proteins. Hub nodes (proteins): red, large size; Term nodes: dark green, large size; other nodes (proteins): yellow, small size; Protein's involvement into biological process: line, dark gray.

In order to find out the potential therapeutic targets for IgG4-RD, we extracted all proteins (not only DEPs) involved in top 30 KEGG/GO BP terms enriched from all DEPs in our tissue proteomic data (Figure 7). One thousand six hundred seventy-four proteins in KEGG top 30 terms and, 2,291 proteins in GO BP top 30 terms were identified. Based on the involvements of these proteins in these different biological processes, we constructed networks and calculated the degree (number of connections, number of pathways in which the protein participates) of each protein. We identified proteins with top 15 degrees as hub proteins. Most of these hub proteins, for example, protein kinase C alpha/beta/delta/gamma type (PRCKA/B/D/G), mitogen-activated protein kinase 1/3 (MAPK1/3), and phosphatidylinositol 4,5-bisphosphate 3-kinase catalytic subunit alpha/beta/delta isoform (PIK3CA/B/D), are important components in immune related signaling pathways in various types of immune cells (27–29). Medication targeting these proteins may help the treatment to IgG4-RD. Therefore, we also obtained the information of targeted drugs to the hub proteins from Therapeutic Target Database (Table 5) (22).

**Table 5:** Potential therapeutic targets and targeted drugs for IgG4-RD.

**Gene symbol**	**Name**	**Drugs (target type)**	**Disease application**
**Targets identified from KEGG terms**			
RAC1	Ras-related C3 botulinum toxin substrate 1	EHT-1864 (Literature-reported target)	Alzheimer disease
ITGB1	Integrin beta-1	131I-radretumab (Clinical trial target)	Non-small-cell lung cancer; Macular degeneration
		ATN-161(Clinical trial Target) *Target: Integrin alpha-5/beta-1	Non-small-cell lung cancer; Renal cell carcinoma
MAPK1	Extracellular signal-regulated kinase 2	CI-1040 (Clinical trial target)	Artery stenosis; Pancreatic cancer
MAPK3	Extracellular signal-regulated kinase 1	BVD-523 (Clinical trial Target)	Solid tumor/cancer; Artery stenosis
PRKCB	Protein kinase C beta	Enzastaurin (Clinical trial target)	Diffuse large B-cell lymphoma; Lymphoma
PRKCA	Protein kinase C alpha	Sodium phenylbutyrate (Successful target)	Spinal muscular atrophy; Renal transplantation;
PIK3CA	Phosphatidylinositol 3-kinase catalytic subunit alpha	BAY 80-6946 (Successful target)	Follicular lymphoma; Non-hodgkin lymphoma
PIK3CB	Phosphatidylinositol 3-kinase catalytic subunit beta	Buparlisib (Clinical trial target)	Breast cancer; Pain
PIK3CD	Phosphatidylinositol 3-kinase catalytic subunit delta	Idelalisib (Successful target)	Follicular lymphoma; Small lymphocytic lymphoma
PRKCG	Protein kinase C gamma	Midostaurin (Successful target)	Acute myeloid leukemia; Systemic mastocytosis
SRC	Proto-oncogene tyrosine-protein kinase Src	Herbimycin A (Successful target)	Breast cancer; Ischemia
**Targets identified from GO Biological process terms**			
NCK1	NCK adaptor protein	AX-024 (Clinical trial target)	Multiple sclerosis
PRKCD	Protein kinase C delta	KAI-9803 (Clinical trial target)	Acute myocardial infarction; Human immunodeficiency virus infection
PTPRC	Receptor-type tyrosine-protein phosphatase C	Iomab-B (Clinical trial target)	Bone marrow transplantation; Acute myeloid leukemia
RAC1	Ras-related C3 botulinum toxin substrate 1	EHT-1864(Literature-reported target)	Alzheimer disease
ABL1	Tyrosine-protein kinase ABL1	Adenosine triphosphate (Successful target)	Breast cancer; Ischemia

*Only available targets were showed*.

## Discussion

To our knowledge, the pathogenesis of IgG4-RD is still not clear and this is the first report providing new insights to help the illustration of potential pathogenic mechanisms underlying IgG4-RD based on proteomic and transcriptomic data.

Existence of strong immune responses in IgG4-RD is well-known. Different T cell subsets may interact with B cell subsets in involved tissue, and further lead to the down-streaming fibrogenesis, which can also be partly reflected by our study (Figure 2) (30–34). In addition, this study indicated that other subclasses of immunoglobulin G could also be pathogenic in IgG4-RD (35). The membrane form of IgG4 is exactly the B cell receptor on IgG4+ plasmablasts/plasma cells, which can mediate the antigen capture and further lead to the antigen presentation from plasmablasts/plasma cells.

Our study detected significant enrichment of “Fc gamma R-mediated phagocytosis” process in IgG4-RD tissue (Figure 2A, Table S7), which may point out the potential role in the pathogenesis of IgG4-RD. Activation of Fcγ receptors (FcγR) on phagocytes can promote phagocytosis and the following antigen presentation process (36). Compared with high binding affinity of IgG1 and IgG3 to FcγR, the binding affinity of IgG4 to FcγR seems to be much milder. In IgG4-RD tissue, our proteomic study showed significant elevated IgG3 level, while IgG1 showed only the tendency of elevation. These clues indicate the potential role of IgG3 in IgG4-RD, at least, partly by Fc gamma receptor mediated phagocytosis, which can be another origin of antigen presentation, together with that mediated by IgG4+ plasmablasts/plama cells.

In this study, besides immunoglobulins produced by plasmablasts/plama cells, we found level of several cytokines, including CXCL13, IL-27, and IL-18, elevated significantly in IgG4-RD tissue. Cytokines exert essential effects on the immunoinflammatory process. IL-27 can act as antagonists to suppress Th1, Th2, Th9, and Th17 responses, while promote the proliferation and the expression of T-bet, EOMES, and IL-12Rβ2 associated with increased production of IFN-γ and cytotoxic activity (37). IL-18 is a member of IL-1 family, and involved not only in Th1 and NK cell activation, but also in Th2, IL-17-producing γδ T cells and macrophage activation (38). CXCL13 is critical for the recruitment of follicular Tfhs, and plasma CXCL13 can be a biomarker for germinal center activity (39, 40). Thus, all these three cytokines may exert important regulatory effects on the immune responses in IgG4-RD. However, these three cytokines were not detected in our serum proteomic analysis, and these might result from their low concentration in serum, and the detection threshold for iTRAQ methods. Thus, more validations should be applied to detect the existence and levels of these cytokines in serum, and examine the availability as biomarkers in IgG4-RD.

Infectious agents can modulate the status of immune responses, and even be the triggers of systemic lupus erythematosus (SLE) and Sjögren's syndrome (41). Our analyses enriched a large amount of processes related to infectious agents in both proteomic (Figure 2A) and transcriptomic data (Tables S7, S8). We may infer that the most frequently involved organs in IgG4-RD including pancreas, salivary glands are mainly exocrine organs, which have more opportunity to contact infectious agents. Activation of toll-like receptors (TLRs) can involve in autoimmune responses indirectly via modulating innate immunity, and directly via modulating B cell signaling, and TLRs can even be the therapeutic targets for autoimmune connective tissue diseases (42, 43). In our proteomic data, the elevation of TLR2 was detected in IgG4-RD samples (Table S1), which indicated the possibility, that TLR2 may have the potential to exert modulatory effects in IgG4-RD *in vivo*.

Enrichment of platelet activation related processes in IgG4-RD patients was also observed in our study (Figures 2, 3, 5). As illustrated above, majority of the recent investigations focus on the pathogenetic roles of traditional immune cells like lymphocytes and macrophages (44). However, immune response is a complex process, and can be modulated by multiple factors. Recently, immunomodulatory effects of platelets have been observed in several immunoinflammatory conditions. Zhu et al. showed platelets first promoted the activation of Th1, Th17, and Treg cells, while only suppressed the immune response of Th1 and Th17 cells secondarily (45). Platelets can also express receptors including FcγRIIA, TLR4, and TLR9, which equips platelets the capacity to receive the stimuli, like immune complexes, PAMPs, and DAMPs, from microenvironments and response to them (46). Granules in platelets contain various growth factors, chemokines, and proinflammatory factors including TGF-β, EGF, CXCL12, HMGB1, and sCD40L etc., which can function in the modulation of immunoinflammatory processes (46, 47).

Genes (or proteins) related to several signaling pathways were also detected in IgG4-RD samples (tissue or PBMC), including MAPK signaling pathway, PI3K-Akt signaling pathway, Ras signaling pathway, TGF-β signaling pathway, NF-κB signaling pathway and Rap1 signaling pathway (Figures 2A, 3, Table 4, Table S7). All these signaling pathway can regulate variety of biological process. Beside biological process described above, genes related to autophagy, necroptosis and adhesion molecules (e.g., “Focal adhesion,” “Cell adhesion molecules (CAMs),” etc.) were also detected in IgG4-RD samples. Interestingly, several terms related to malignancy (e.g., “Choline metabolism in cancer,” “Small cell lung cancer,” “Bladder cancer,” “Viral carcinogenesis,” and “Proteoglycans in cancer”) were also enriched in IgG4-RD samples. All these clues can reflect some parts of the characteristics of IgG4-RD, and indicate the potential similarities of the pathogenesis mechanisms underlying IgG4-RD to the known mechanisms of the biological processes illustrated above.

How to treat IgG4-RD is another task we are facing. Despite glucocorticoids and rituximab are effective in the induction therapy, and application of conventional steroid sparing medication (e.g., azathioprine, mycophenolate mofetil, and methotrexate) can help control the disease, the problem of relapse and side effects after long-term usage of these medications are still remarkable (48–50). Based on the characteristics of IgG4-RD tissue proteomic data, we identified several hub proteins, which might be involved and play important roles in the pathogenesis of IgG4-RD. Most of these proteins are important signaling components in immune reactions, and some of these agents have been even applied into the clinical treatment of other diseases. Therefore, after the validation of the pathogenic roles of these proteins in IgG4-RD via laboratory experiments, these drugs have the potential to be therapeutic agents targeting IgG4-RD.

In summary, we provided the first integrative analysis of IgG4-RD via both proteomic and transcriptomic data, and described a landscape of biological processes of this mysterious disease, which indicated some potential pathogenic molecules and immunoinflammatory responses, and provided several potential therapeutic targets for the treatment of IgG4-RD. There are also limitations in our study. Firstly, despite the aim of our study is to provide the landscape at the level of mRNA and protein of IgG4-RD, the sample size of our analysis was relatively small, and we didn't enroll more IgG4-RD-like samples (e.g., tumors, and other rheumatic diseases), in our analyses; secondly, the transcriptomic data were originated from the published data of other centers, although analyses to them showed overlaps with our proteomic data, which may reflect the reliability of our analyses indirectly; thirdly, our analyses were based on the data of bulk samples, which cannot provide the information of specific cell types. Therefore, further studies with large sample size, and at single cell level are needed. Besides, other types of omics (e.g., lipidomics, metabolomics, and glycomics) and validation with laboratory experiments are also important to help us understand this mysterious disease more deeply.

## Data Availability Statement

Data of GSE40568 and GSE66465 can be downloaded from Gene Expression Omnibus Dataset (GEO Dataset: http://www.ncbi.nlm.nih.gov/geo/). The proteomic data is available from the corresponding author on reasonable request.

## Ethics Statement

The studies involving human participants were reviewed and approved by Tongji Hospital, Tongji Medical College, Huazhong University of Science, and Technology Institutional Review Board Approval. The ethics IRB ID is: TJ-C20151109. The patients/participants provided their written informed consent to participate in this study.

## Author Contributions

SC and YC made the sample collection, data analyses, and wrote this manuscript. SL and CY revised the manuscript and provided important advice. LD and FZ designed this program, and are the corresponding authors of this paper. All authors contributed to the article and approved the submitted version.

## Conflict of Interest

The authors declare that the research was conducted in the absence of any commercial or financial relationships that could be construed as a potential conflict of interest.

## Supplementary Material

Figure S1 Scale-free topological network of IgG4-RD LSG samples in GSE40568 dataset by WGCNA. Scale independence (Left) and mean connectivity (Right) were plotted, respectively.

Figure S2 Scale-free topological network of IgG4-RD PBMC samples in GSE66465 dataset by WGCNA. Scale independence (Left) and mean connectivity (Right) were plotted, respectively.

Figure S3 Module membership plots of “turquoise” **(A)** module and **(B)** eigengenenetwork plots in GSE40568.

Figure S4 Module membership plots of “yellow” **(A)** module and **(B)** eigengenenetwork plots in GSE66465.

Table S1 Differentially expressed proteins between IgG4-RD patients and controls in tissue.

Table S2 Differentially expressed proteins between IgG4-RD patients and controls in serum.

Table S3 Cytoscape Input nodes in turquoise.

Table S4 Cytoscape Input edges in turquoise.

Table S5 Cytoscape Input nodes in yellow.

Table S6 Cytoscape Input edges in yellow.

Table S7 KEGG analysis with genes (adjacency value > 0.2) in turquoise module in tissue transcriptomic data.

Table S8 KEGG analysis with genes (adjacency value > 0.2) in yellow module in PBMC transcriptomic data.

## Abbreviations

IgG4: Immunoglobulin G4
iTRAQ: Isobaric tags for relative and absolute quantitation
CV: coefficient of variation
WGCNA: weighted gene co-expression network analysis
KEGG: Kyoto Encyclopedia of Genes and Genomes
DEP: differentially expressed protein
FDR: false discovery rate
LSG: labial salivary glands
PBMC: peripheral blood mononuclear cell.
BCR: B cell receptor
PAMP: pathogen associated molecular patterns
DAMP: danger associated molecular patterns.
PRR: pattern recognition receptor
MMP: matrix metalloproteinase.
