# Supplementary figures and images for: Multiple Processes May Involve in the IgG4-RD Pathogenesis: An Integrative Study via Proteomic and Transcriptomic Analysis
Source: Front Immunol. 2020 Aug 20;11:1795. doi: 10.3389/fimmu.2020.01795 (PMC7468437; doi:10.3389/fimmu.2020.01795)

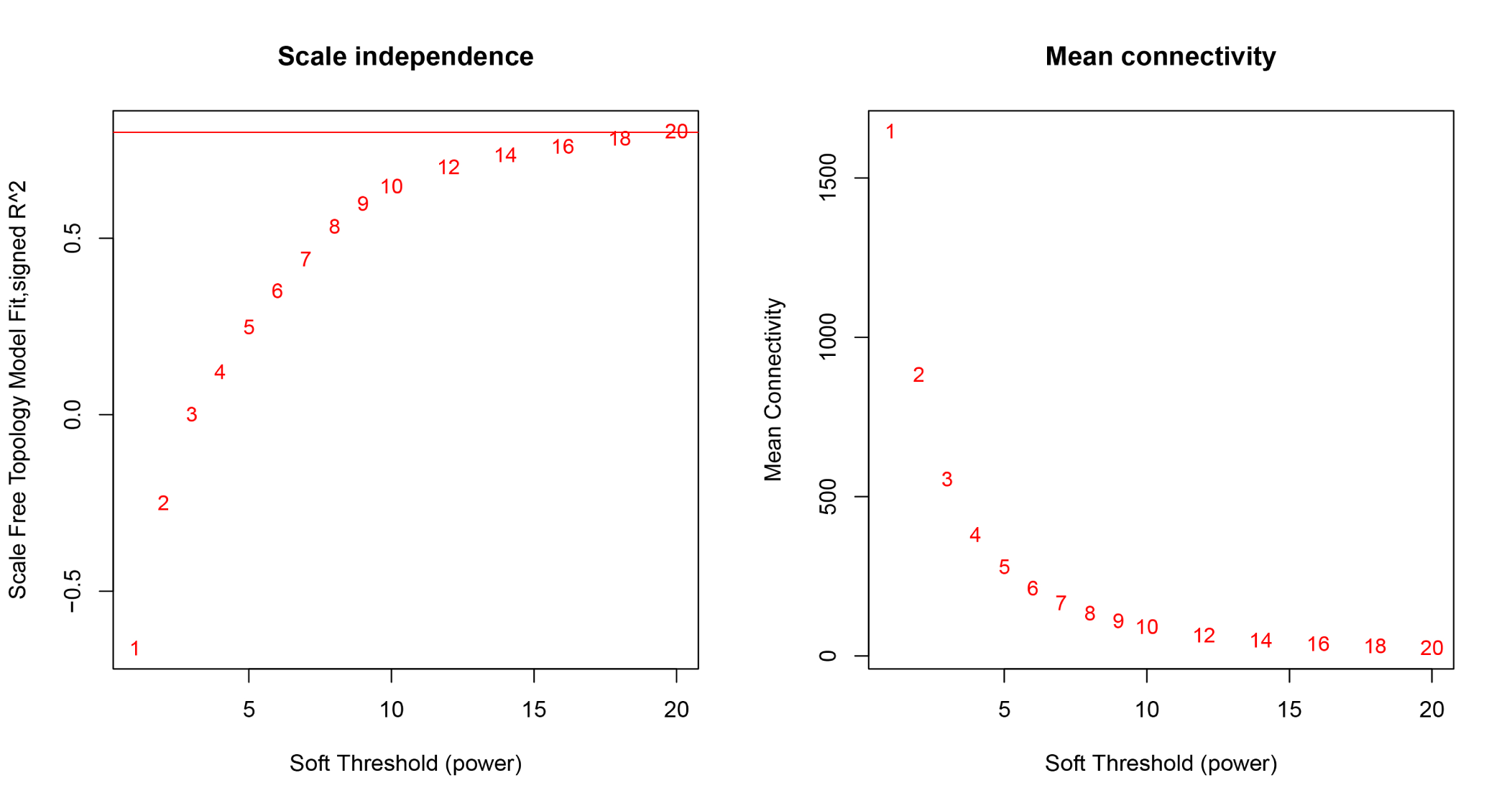

Supplement: Figure S1 — Scale-free topological network of IgG4-RD LSG samples in GSE40568 dataset by WGCNA. Scale independence (Left) and mean connectivity (Right) were plotted, respectively. [file Image_1.TIF]

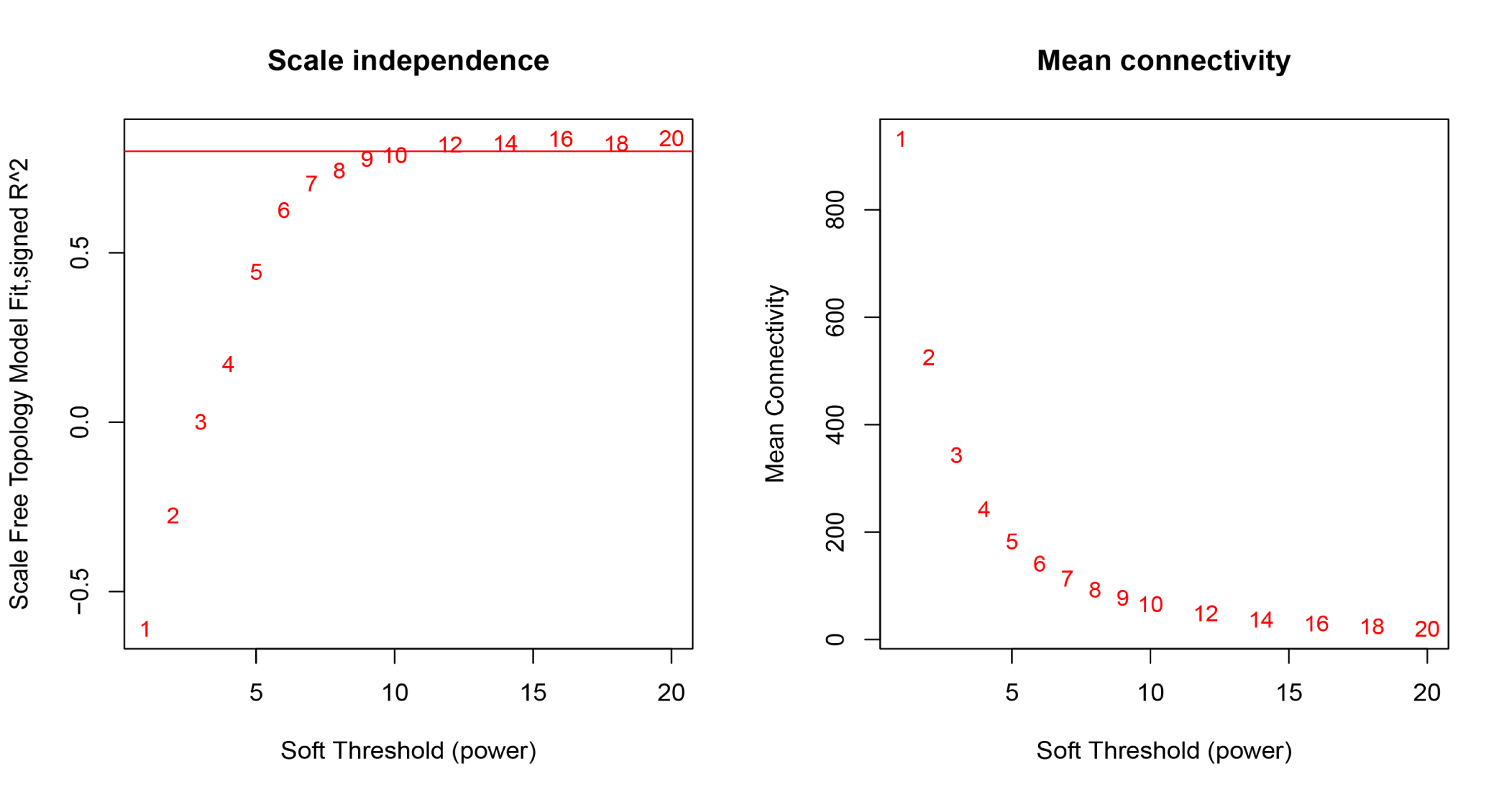

Supplement: Figure S2 — Scale-free topological network of IgG4-RD PBMC samples in GSE66465 dataset by WGCNA. Scale independence (Left) and mean connectivity (Right) were plotted, respectively. [file Image_2.TIF]

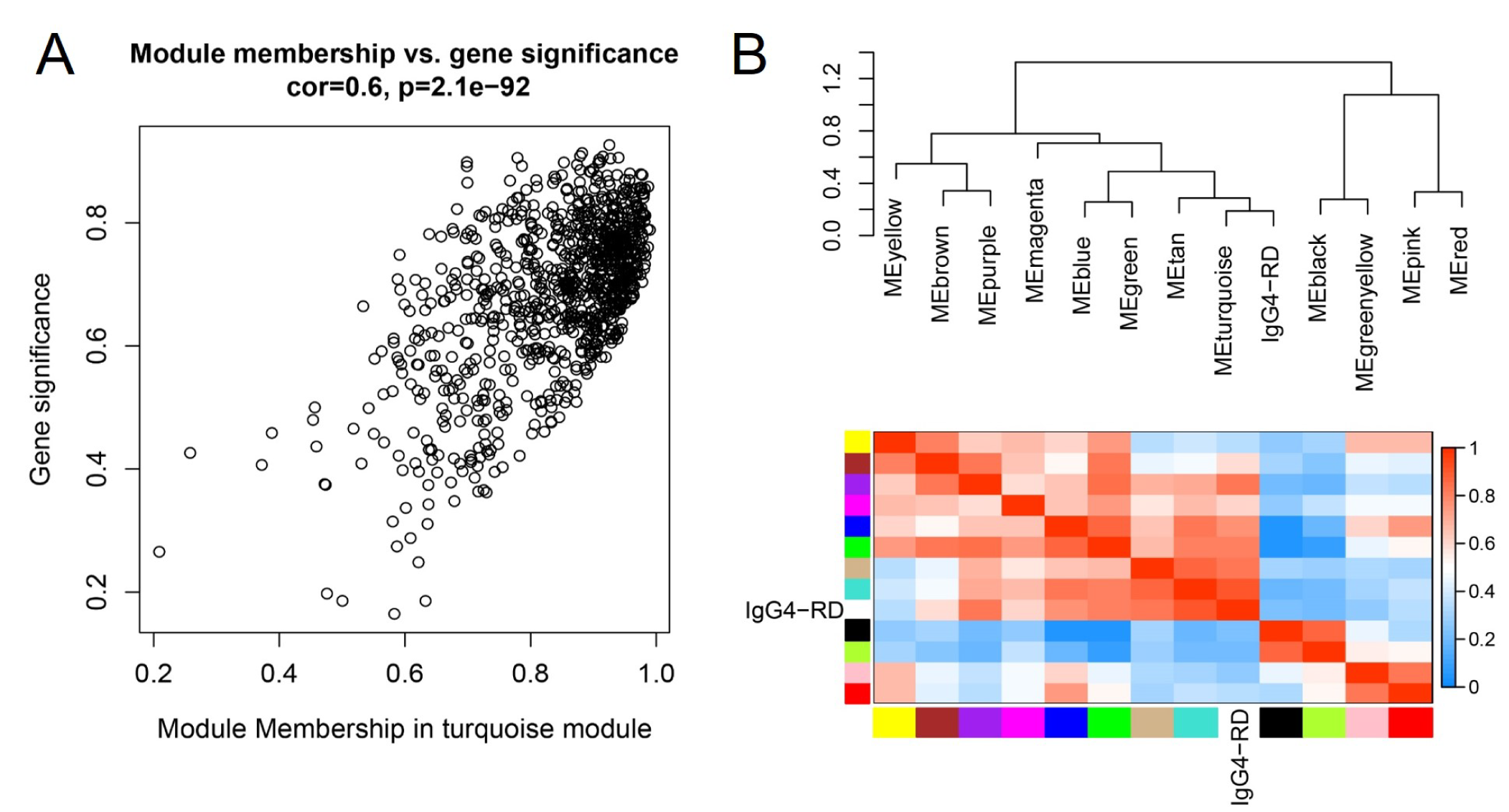

Supplement: Figure S3 — Module membership plots of “turquoise” (A) module and (B) eigengenenetwork plots in GSE40568. [file Image_3.TIF]

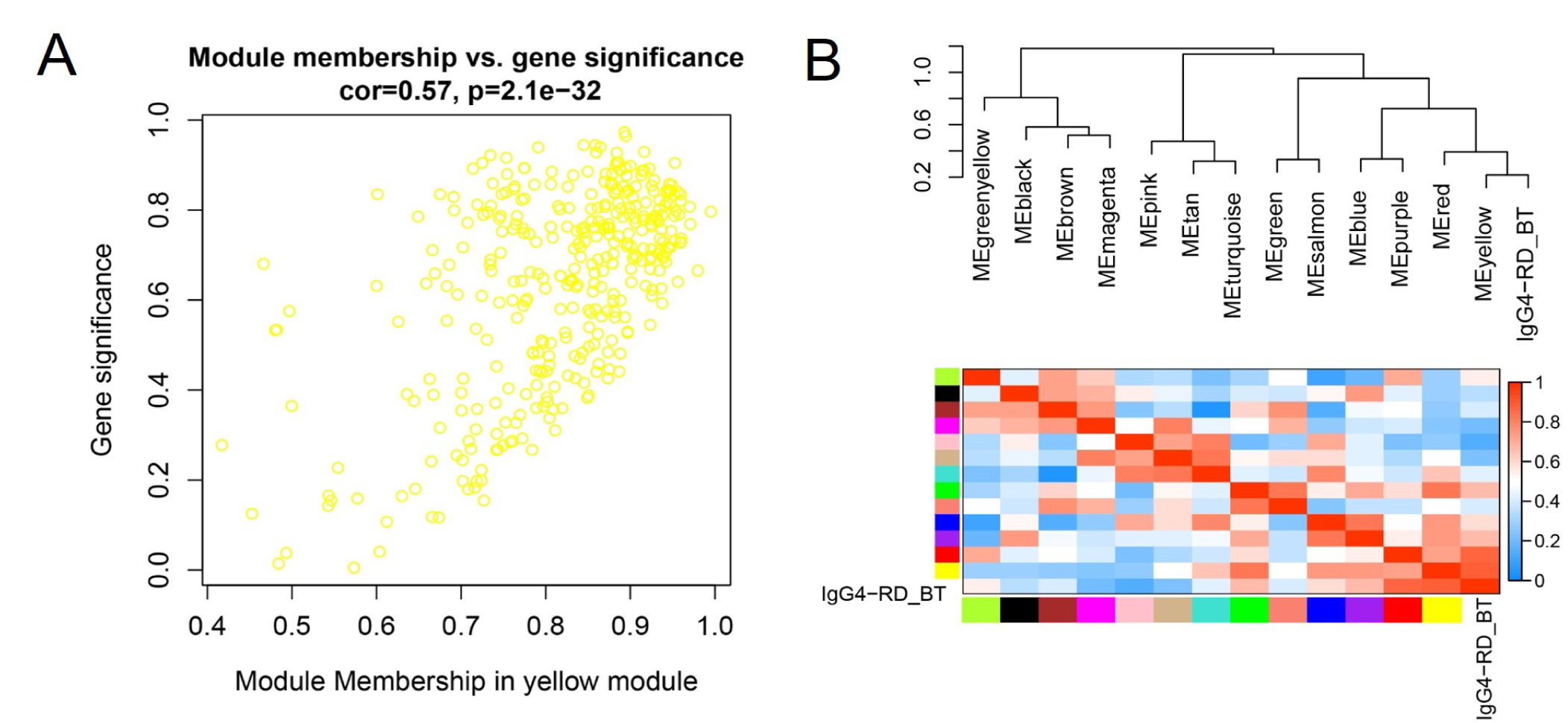

Supplement: Figure S4 — Module membership plots of “yellow” (A) module and (B) eigengenenetwork plots in GSE66465. [file Image_4.TIF]
